# Synergetic reorganization of the contralateral structure and function in patients with unilateral frontal glioma

**DOI:** 10.3389/fnins.2022.1016693

**Published:** 2022-09-23

**Authors:** Honglin Ge, Zheng Yan, Dongming Liu, Wenzhang Qi, Shanshan Chen, Kun Yang, Hongyi Liu, Yuanjie Zou, Xinhua Hu, Yong Liu, Jiu Chen

**Affiliations:** ^1^Department of Neurosurgery, The Affiliated Brain Hospital of Nanjing Medical University, Nanjing, China; ^2^Department of Radiology, The Affiliated Brain Hospital of Nanjing Medical University, Nanjing, China; ^3^Department of Neurology, The Affiliated Brain Hospital of Nanjing Medical University, Nanjing, China; ^4^Institute of Neuropsychiatry, The Affiliated Brain Hospital of Nanjing Medical University, Nanjing, China; ^5^Institute of Brain Functional Imaging, Nanjing Medical University, Nanjing, China

**Keywords:** resting-state fMRI, voxel-based morphometry, frontal glioma, plasticity, contralateral reorganization

## Abstract

**Objective:**

This study aimed to investigate the contralateral structural and functional plasticity induced by frontal gliomas.

**Methods:**

Patients with left (*n* = 49) or right (*n* = 52) frontal diffuse glioma were enrolled along with 35 age- matched healthy controls (HCs). The gray-matter volumes (GMVs) of the contralesional region were measured using the voxel-based morphometry (VBM) analysis. Additionally, the amplitude of low-frequency fluctuation (ALFF) of the contralesional region was calculated via resting state functional magnetic resonance imaging (MRI) to assess functional alterations.

**Result:**

The GMV of the contralateral orbitofrontal cortex of the right or left frontal gliomas was significantly larger than the corresponding GMV in the controls. In the patients with right frontal glioma, the GMV and ALFF in the left inferior frontal gyrus were significantly increased compared with those in the controls.

**Conclusion:**

Glioma invasion of the frontal lobe can induce contralateral structural compensation and functional compensation, which show synergy in the left inferior frontal gyrus. Our findings explain why patients with unilateral frontal glioma can have functional balance, and offer the possibility of preserving the brain function while maximizing tumor removal.

## Introduction

Glioma accounts for > 70% of malignant brain tumors (Ostrom et al., 2020) and is known for its strong invasiveness, rapid cell proliferation, and angiogenesis (Angelopoulou and Piperi, 2018). The frontal lobe, one of the most frequently invaded areas by glioma (Ostrom et al., 2020), is involved in a range of functions, including language (Wang et al., 2020), cognitive functions (Li et al., 2013), emotions, executive functions (Hiser and Koenigs, 2018; Rudebeck and Rich, 2018; Rolls, 2019), and motor functions (Krauzlis, 2002). A local frontal lesion can impair the functions corresponding to the injured region (Cai et al., 2016; Stockert et al., 2020). However, clinical observations show that frontal glioma is not accompanied by the expected widespread functional deficits, even if the tumor invades important functional areas. Thus, some type of compensation seems to occur in the brain to adapt to the effects of glioma.

Plasticity is described as the ability of the brain to adapt to changes in the external environment and internal needs. Plasticity is implemented by functional and structural reorganization of the brain (Almairac et al., 2018; Liu et al., 2020). In this case, the brain rearranges redundant and latent networks to compensate for the loss of function caused by the injury (Herbet et al., 2016). Such rearrangement may occur around the lesion, ipsilateral and contralateral hemispheres of the lesion, and cerebellum (Heiss et al., 2003; Almairac et al., 2018; Zhang et al., 2018). Frontal reorganization has previously been verified (Cai et al., 2016; Stockert et al., 2020), and contralateral compensation has also been demonstrated in temporal and insular gliomas (Almairac et al., 2018; Liu et al., 2020). However, the contralateral compensation in frontal gliomas has received little attention, and structural reorganization was analyzed separately from functional reorganization. In fact, structural reorganization and functional reorganization share a common physiological mechanism (May et al., 2007; Gupta et al., 2018). Therefore, it is necessary to study the synergism between the contralateral structural compensation and functional compensation in frontal glioma.

Voxel-based morphometry (VBM) is a widely used automated technique to analyze results from structural magnetic resonance imaging (MRI) and can identify regional differences in relative gray matter (GM) (Almairac et al., 2018). Functional MRI (fMRI) is an imaging method based on signals dependent on blood oxygenation level. These signals are used to generate maps that reflect the actual neural changes, and help us to discern the elementary units of the activated networks and the time course of various neural events (Logothetis, 2008). fMRI has been widely used to explore functional reorganization of the brain (Grefkes and Ward, 2014; Miotto et al., 2014; Stockert et al., 2020). The intensity of intrinsic spontaneous neural activity can be expressed as the amplitude of low-frequency fluctuations (ALFFs) (Gupta et al., 2018), which can reliably and intuitively reflect the functional compensation.

To sum up, we used VBM and ALFF to assess the contralateral structural and functional reorganization in patients with frontal glioma. We hypothesized that lesions invading the frontal lobe would induce structural and functional reorganization of the contralesional region. We also hypothesized that the two types of reorganization may have some synergy. This structural and functional reorganization may protect patients with unilateral glioma from the harmful effects of the tumor invasion into the frontal lobe.

## Materials and methods

### Participants

**FIGURE 1:**
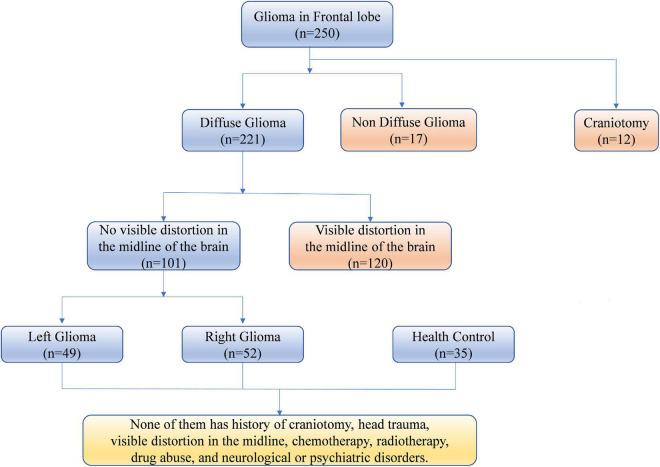
Patient screening.

We included 101 patients with frontal lobe diffuse gliomas who underwent surgical resection at Nanjing Brain Hospital between June 2013 and February 2022. The study was approved by the ethics committee of Nanjing Brain Hospital, and all patients provided written informed consent. This cohort included 49 patients with left frontal glioma [mean age 52.84 ± 12.11 (24–77) years; 25 women, mean age 52.00 ± 13.22 (24–75); and 24 men, mean age 53.71 ± 10.77 (34–77)] and 52 patients with right frontal glioma [mean age 50.96 ± 14.36 (26–86) years; 21 women, mean age 54.90 ± 14.93 (27–86); and 31 men, mean age 48.29 ± 13.31 (26–67)]. 11 patients with right frontal gliomas also had invasion of the right insula, while 10 patients with left frontal gliomas also had invasion left insula. The flowchart in Figure 1 summarizes the process of patient screening.

### Inclusion and exclusion criteria

(1) Localized gliomas (mainly pilocytic astrocytomas) were excluded to improve the overall homogeneity, due to their different biological specificities (e.g., growth and invasiveness kinetics) from diffuse gliomas (Seifert et al., 2015). (2) Any patient who had undergone craniotomy was excluded to avoid influence of surgery. (3) The imaging data of each patient were checked to ensure that no midline structure, including the septum pellucidum, corpus callosum, and third ventricle, was distorted. (4) No patient had a history of head trauma, chemotherapy, radiotherapy, drug abuse, or neurological or psychiatric disorder.

The same criteria were applied to the 35 healthy controls [HCs; 27 women and 8 men; mean age 54.94 ± 5.02 (42–60) years], recruited from the general community.

### Magnetic resonance imaging data acquisition

All the MRI images were acquired on a 3.0-T Verio Siemens Scanner equipped with an eight−channel phased−array head radiofrequency coil at the Department of Radiology, the Affiliated Brain Hospital of Nanjing Medical University.

The structural scans of the 101 patients and 35 HCs were axially acquired using high-resolution three-dimensional (3D) T1-weighted magnetization-prepared rapid gradient echo with the following acquisition parameters: matrix = 256 × 256, echo time (TE) = 2.49ms, repetition time (TR) = 1.9 s, time inversion (TI) = 900ms, flip angle (FA) = 9, slice thickness = 1mm, gap = 0.5mm, and slice number = 176 slices covering the whole brain.

Of the 101 patients, 36 had resting-state fMRI (rsfMRI) images, which had been obtained using a gradient-recalled echo-planar imaging sequence, including 140/240 volumes. The parameters of 140 volumes rsfMRI (the scan time was from 2013 to 2016) are as follow: TRs = 2,000 ms, TEs = 30 ms, FAs = 90°, acquisition matrices = 64 × 64, fields of view (FOV) = 240 mm × 240 mm, thicknesses = 3.0 mm, gaps = 4 mm, numbers of slices = 30, and voxel sizes = 3.75 × 3.75 × 4 mm^3^. The parameters of 240 volumes rsfMRI (the scan time was from 2017 to 2022) are as follow: TRs = 2,000 ms, TEs = 30 ms, FAs = 90°, acquisition matrices = 64 × 64, FOV = 220 mm × 220 mm, thicknesses = 4.0 mm, gaps = 0 mm, numbers of slices = 36, and voxel sizes = 3.4 × 3.4 × 4 mm^3^.

Different parameters had been used to optimize and improve the imaging protocol, and they were not related to the purposes of this study. Even if the parameters were homogeneous in the same scanner, parameter differences were included in the statistical analysis as a covariable of non-interest.

### Image preprocessing of resting-state functional magnetic resonance imaging

The MRI images were preprocessed using the Data Processing and Analysis for Brain Imaging (DPABI)^1^ toolkit (Yan et al., 2016) and the Statistical Parametric Mapping 12 (SPM12)^2^ toolkit in MATLAB (release 2013b).^3^

The first five functional volumes were excluded from each subject to remove mechanical noise at the start of the scan. Images were separately slice-timed and rearranged to correct for the temporal differences and head motion in the remaining images. The criterion of head motion was set as 3 mm in translation and 3° in rotation (Zhang et al., 2018). The structural images (T1-weighted) and fMRI were subsequently manually reoriented and shifted, manually selecting the anterior commissure as the origin (coordinates 0, 0, 0). A single T1 image was uniformly segmented; the obtained functional image was spatially normalized to the standard MNI space, and the normalized image was resampled to an isotropic resolution of 3 × 3 × 3 mm^3^. Regressions were performed on common interfering signals, including Friston’s 24 head motion parameter (Karl et al., 1996), white matter (WM), and cerebrospinal fluid (CSF) signals. Due to the ambiguity regarding the regression of the global signal (Zhang et al., 2018), this variable was not regressed out. After this, the detrend procedure was applied to the fMRI data. The resulting images were smoothened using 8-mm full width at half-maximum Gaussian smoothing.

### Gray matter analysis

To explore the contralateral frontal lobe for any structural change, the VBM method was used as follows: (1) Each image was segmented into GM, WM, and CSF by using SPM12. (2) The Diffeomorphic Anatomical Registration Through Exponentiated Lie Algebra algorithm was then used to spatially normalize the segmented images (Ashburner and Friston, 2009). (3) By running the internal code in MATLAB, the total intracranial volume (TIV) of each patient and HC was calculated according to GM, WM, and CSF. (4) The segmented tissues were projected into MNI space via affine transformation. (5) The obtained images were smoothened using a Gaussian kernel with an FWHM of 8mm. The final GM images, which were normalized, modulated, and smoothened, were subjected to the following statistical analyses.

### Spontaneous neural activity analysis

ALFF can effectively reflect the intensity of spontaneous neural activity (Gupta et al., 2018). The time series for each voxel was transformed to the frequency domain, and the power spectral density was obtained. Since the power of the given frequency is proportional to the square of the respective amplitude, the square root can be calculated on each frequency of the power spectrum. The mean square root of each voxel was obtained in the range of 0.01–0.1 Hz as the ALFF of the voxel. The ALFF value of each voxel point was calculated using DPABI and divided by the mean value of the whole brain to obtain the mean amplitude of low-frequency fluctuation (mALFF) value, which was subjected to the following statistical analyses.

### Mask fabrication

It should be pointed out that few gliomas can grow in isolation in a single lobe, and gliomas in the frontal lobe often involve the insula. Therefore, we set the GM of the frontal lobe and insula as the region of interest (ROI) and used the WFU-Pickatlas tool-box for SPM12^4^ to generate masks.

### Statistical analysis

We compared the GMV between the contralateral frontal lobe and insula with the corresponding position in the HCs by using the Resting State fMRI Data Analysis Toolkit with the two-sample *t-*test, and the results were corrected using the threshold-free cluster enhancement family-wise error (TFCE-FWE) correction. To verify that this change was specific to the ROI, we compared the GMVs between the superior occipital gyrus (SOG) in the contralateral hemisphere of the glioma with the corresponding position in the HCs. The SOG was chosen because it is far from the frontal lobe and is not easily disturbed by lesions. The GMs of the SOG and the regions with significant differences were extracted and their volumes were presented as bar charts.

Similar to GMV, the mALFF values of the patients were compared with those of the controls by using the two-sample *t*-test, and the results were also corrected using TFCE-FWE. Then, the mALFF values of the SOG and the regions with significant differences were presented as bar charts.

Statistical Product Service Solutions was used to analyze the demographic data, GMVs, and mALFF values. One-way analysis of variance (ANOVA) was used to compare the TIVs and age ranges of different groups. The Chi-square test was used to compare gender ratios. The cluster size > 20 voxels was applied for further non-parametric replacement test, which was used to compare the GMVs and mALFF values of the patients with those of the HCs by using the DPABI toolkit. The TFCE-FWE–corrected cluster *P*-value < 0.05 was considered to indicate statistical significance. The permutation times were set at 1,000 tests. TIV, age, and gender were treated as covariables in the statistical analysis of GMV and mALFF.

## Results

### Demographic and clinical characteristics

The demographic data and clinical characteristics of all the participants are shown in Table 1. All the patients and HCs were of Han Chinese descent and right-handed according to the Edinburgh Handedness Inventory.

**TABLE 1:** Demographic and clinical characteristics.

Variable	HCs	Structural MRI		*P*-value	Functional MRI		*P-*value
		Left frontal	Right frontal		Left frontal	Right frontal	
Gender female/male^†^	27/8	25/24	21/31	0.02 *	3/12	4/17	0.000 ****
WHO grade II/III/Iv^†^	NA	26/5/18	26/8/18	NA	7/2/6	10/3/8	NA
Age (year) total	54.94 ± 5.02 [42–60]	52.84 ± 12.11 [24–77]	50.96 ± 14.36 [26–86]	0.31	54.87 ± 11.79 [34–77]	51.05 ± 11.99 [28–74]	0.29
Female	54.22 ± 5.32 [42–60]	52.00 ± 13.22 [24–75]	54.90 ± 14.93 [27–86]	NA	48.00 ± 6.48 [42–57]	55.50 ± 13.87 [35–74]	NA
Male	57.36 ± 2.64 [52–60]	53.71 ± 10.77 [34–77]	48.29 ± 13.31 [26–67]	NA	56.58 ± 12.18 [34–77]	50.00 ± 11.25 [28–68]	NA
Handedness L/R/A^†^	0/35/0	0/49/0	0/52/0	NA	0/15/0	0/21/0	NA
TIV (cm^3^)	1309.30 ± 117.01 [1063.61–1485.02]	1300.04 ± 123.93 [859.36–1545.94]	1311.01 ± 132.23 [1068.30–1604.66]	0.90	NA	NA	NA
Functional MRI volumes 140/240^†^	0/35	NA	NA	NA	7/8	7/14	NA

*P*-value was calculated using the Chi-square test for the gender, and one-way ANOVA for the age and TIV. ^†^Values are number of patients. *P-value < 0.05, ****P-value < 0.0001. HC, Healthy Control; NA, Not Applicable; L/R/A, Left/Right/Ambidextrous; TIV, Total Intracranial Volume; MRI, Magnetic Resonance Imaging.

There was no difference in age or TIV between the patients and HCs, but the two groups differed in gender. The number of WHO grade II/III/IV in the left frontal lobe was 26/5/18 (in rsfMRI, 7/2/6), and on the right side was 26/8/18 (in rsfMRI, 11/3/8).

### Changes in contralateral gray-matter volume

**FIGURE 2:**
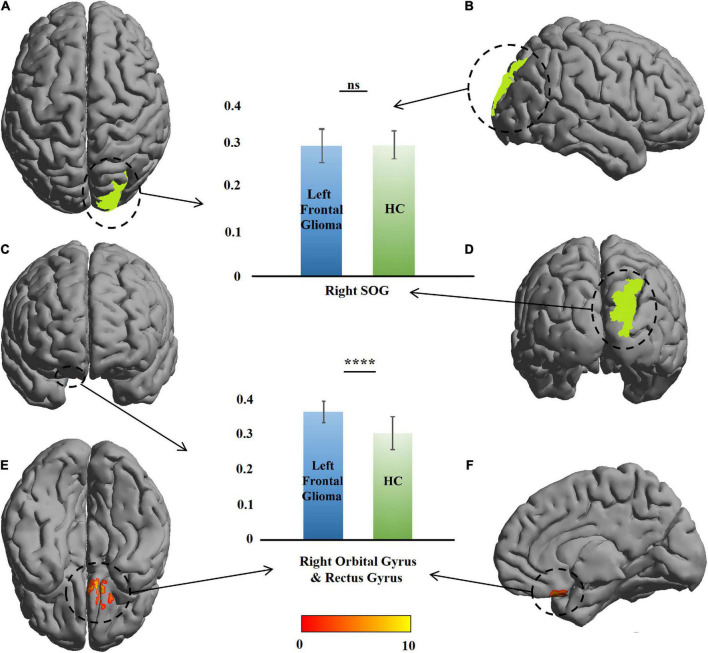
Comparison of gray-matter volume (GMV) between the patients with left frontal glioma and healthy controls (HCs). The warm colors (red to yellow) indicate the significant area (the right orbital gyrus and gyrus rectus) with the right frontal GM indicated as the mask. Green color indicates the right superior occipital gyrus (SOG). The GMVs of the patients and HCs are presented as histograms. ns, not significant. ****P ≤ 0.0001. **(A)** Top view; **(B)** right view; **(C)** anterior view; **(D)** posterior view; **(E)** bottom view; **(F)** left view (only right cerebral hemisphere).

VBM revealed a GMV increase of 442 voxels in the GM of the right orbital gyrus (ROrG) and right gyrus rectus (RGR) in the left-glioma group (*n* = 49), compared with the level in the HCs. There were three voxel peaks—(*x* = 10.5, *y* = 2, *z* = –28.5), (*x* = 9, *y* = 70.5, *z* = 15), and (*x* = 6, *y* = 58.5, *z* = 37.5) In the significant region, the GMV of the patients were larger than that of the HCs (*t* = 7.08, *P* = 7.01 × 10^–10^), and no change was found in the right SOG (*t* = 0.65, *P* = 0.51) (Table 2 and Figure 2).

**TABLE 2:** Analysis of structural and function between the patients and healthy controls.

GMV or ALFF	Group	Region	Peak MNI coordinate	Cluster size (in voxel)	T score peak level
GMV	Contralateral of the left frontal glioma	GR and OrG	(10.5, 2, –28.5)	274	9.3291
GMV	Contralateral of the right frontal glioma	GR and OrG	(–15, 22.5, –28.5)	176	7.8741
			(–27, 36, –22.5)	42	6.6694
		IFG and MFG	(–55.5, 18, –1.5)	774	6.945
			(–49.5, 42, 18)	494	6.5014
ALFF	Contralateral of the right frontal glioma	IFG and Insula	(–48, 12, 0)	54	5.6271

The table shows the clusters whose *P* < 0.05 after TFCE-FWE correction. MNI, Montreal Neurological Institute; GMV, Gray Matter Volume; ALFF, amplitude of low-frequency fluctuation; GR, Gyrus Rectus; OrG, Orbital Gyrus; IFG, Inferior Frontal Gyrus; MFG, Middle Frontal Gyrus.

**FIGURE 3:**
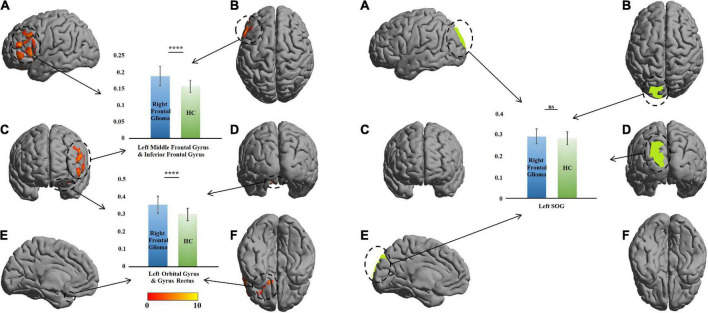
Comparison of gray-matter volume (GMV) between the patients with right frontal glioma and healthy controls (HCs). The warm colors (red to yellow) indicate the significant area (the right orbital gyrus and gyrus rectus) with the right frontal GM indicated as the mask. Green color indicates the right superior occipital gyrus (SOG). The GMVs of the patients and HCs are presented as histograms. ns, not significant. ****P ≤ 0.0001. **(A)** left view; **(B)** top view; **(C)**, anterior view; **(D)** posterior view; **(E)** right view (only left cerebral hemisphere); **(F)** bottom view.

A similar change was found in the right-glioma group (*n* = 52). VBM revealed a GMV increase of 218 voxels in the GM of the left orbital gyrus (LOrG) and left gyrus rectus (LGR). The voxel peaks were located on (*x* = –15, *y* = 22.5, *z* = –28.5) and (*x* = –27, *y* = 36, *z* = –22.5). Different from the left-glioma group, the left middle frontal gyrus (LMFG) and left inferior frontal gyrus (LIFG) were also found to have a GMV increase of 1,268 voxels. The voxel peaks were located on (*x* = –55.5, *y* = 18, *z* = –1.5) and (*x* = –49.5, *y* = 42, *z* = 18). In the significant region, the GMV of the patients was larger than that of the HCs (*t* = 5.62, *P* = 3.88 × 10^–8^ in the LMFG and LIFG; *t* = 5.82, *P* = 2.55 × 10^–8^), and no change was found in the left SOG (*t* = 1.09, *P* = 0.27) (Table 2 and Figure 3).

### Changes in contralateral mean amplitude of low-frequency fluctuation

**FIGURE 4:**
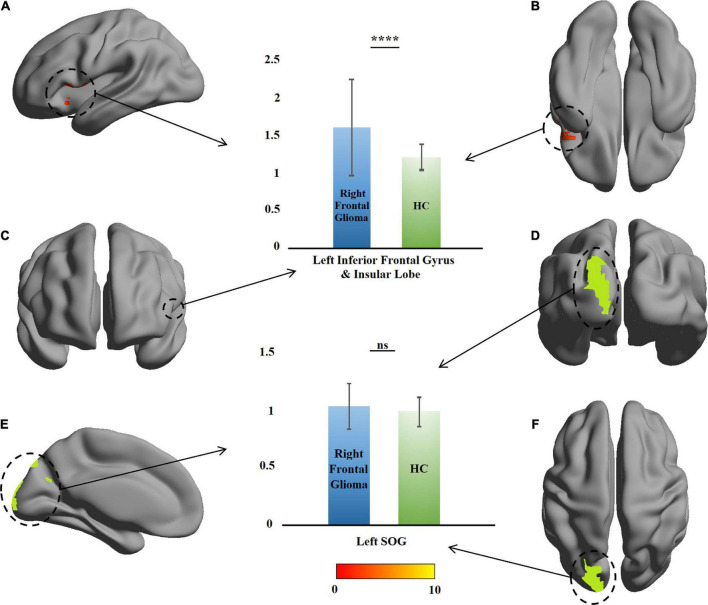
Comparison of amplitude of low-frequency fluctuation between the patients with right frontal glioma and healthy controls (HCs). The warm colors (red to yellow) indicate the significant area (the right orbital gyrus and gyrus rectus) with the right frontal mean amplitude of low-frequency fluctuation (mALFF) indicated as the mask. Green color indicates the right superior occipital gyrus (SOG). The mALFF of the patients and HCs are presented as histograms. ns, not significant. ****P ≤ 0.0001. **(A)** Left view; **(B)** bottom view; **(C)** anterior view; **(D)** posterior view; **(E)** right view (only left cerebral hemisphere); **(F)** top view.

Compared with the HCs, no change was found in the ROI of the left-glioma group (*n* = 15). In the right-glioma group (*n* = 21), an mALFF value increase of 54 voxels was found in the LIFG and left insula. The voxel peak was located on (–48, 12, 0), close to one of the voxel peaks of the GMVs. In the significant region, the mALFF value of the patients was higher than that of the HCs (*t* = 3.43, *P* = 0.001), and no change was found in the left SOG (*t* = 1.52, *P* = 0.19) (Table 2 and Figure 4).

## Discussion

This study is the first to explore the contralateral plasticity in frontal gliomas by using a large sample size (*n* = 101). We combined the strengths of fMRI and structural MRI to evaluate contralateral functional and macrostructural plasticity in patients with frontal gliomas. Our results suggest that both structural compensation and functional compensation of LIFG (primarily Broca’s area) occur when glioma invades the right frontal lobe. Additionally, the contralateral OrG and GR (i.e., the orbitofrontal cortex, OFC) undergo structural compensation regardless of whether the tumor invaded the left or right frontal lobe. This study demonstrates that LIFG and OFC are the main contralateral compensation sites for glioma invasion of the frontal lobe, which explains the functional balance in patients with unilateral frontal glioma. In fact, the purpose of surgery is to achieve “onco-functional balance.” Performing surgery in a compensable area facilitates maximum tumor resection with minimal neurological impairment (Duffau and Mandonnet, 2013). Our work demonstrates the contralateral plasticity in patients with frontal glioma, which might provide evidence for the function protection when preform iterative or maximum tumor resection.

The brain can compensate for structural damage caused by various factors, such as tumors, by reprogramming its survival network (Grefkes and Ward, 2014; Herbet et al., 2016). The findings of the present study showed that unilateral frontal tumors cause contralateral structural and functional recombination, mainly in the LIFG and OFC, mediated by transcallosal disinhibition and increased anisotropy in the corpus callosum (Heiss et al., 2003; Tantillo et al., 2016). Structural reorganization is considered to reflect increased cell size, spine density, or neural or glial cell genesis (May et al., 2007; Ceko et al., 2013), and at the macro level, it is reflected by GMV increase. The microscopic functional reorganization is directly reflected by changes in ALFF (Gupta et al., 2018). The basis for this interhemispheric reorganization probably stems from a networked brain, which allows the effects of a disease to be transmitted not just in short distances but to far regions of the brain (Zhang et al., 2018; Stockert et al., 2020). In short, our structural and functional analyses here confirmed the high level of plasticity in the insula of glioma patients (Almairac et al., 2018) and additionally showed that other areas in the brain, such as LIFG and OFC, have high plasticity in glioma patients, thereby providing new evidence for functional compensation in the brain.

We observed that when a glioma invades the right frontal lobe, both structural compensation and functional compensation occurred in the left IFG. This structural and functional synergism highlights the importance of the Broca’s area in the frontal compensation, and this area may be the functional compensation center of the frontal lobe. The plasticity of the IFG has been confirmed in many studies (Cai et al., 2016; Krishna et al., 2021). This plasticity may result from a combination of two mechanisms: 1. Spare functional capacity, which is suppressed under the physiological conditions but induced after brain injury to compensate for the impaired function; 2. Other networks, after the original functional network is damaged, can adapt and perform the original functions of the damaged network (Stefaniak et al., 2021). The Broca’s area performs crucial language functions, and in our hypothesis, this functional importance enables this area to have considerable functional redundancy to undertake compensatory tasks when glioma invades the frontal lobe, and thus structural reorganization and functional reorganization occur synergetically.

The main functions of the LIFG and right inferior frontal glioma (RIFG) are not the same. The LIFG is involved in the processing of many language subdomains, including object-naming and semantic recognition (Wang et al., 2020; Xu et al., 2020). The LIFG is also thought to be concerned with the processing of working memory and empathy (Liakakis et al., 2011). The RIFG, by contrast, is primarily associated with emotions (Drobinin et al., 2019; Hartwigsen et al., 2019) but also with specific language functions (Gao et al., 2020). In the present study, the LIFG and RIFG showed different results. In our hypothesis, like functional asymmetry, there is also asymmetry in the plasticity of the IFG. This is not to say that the RIFG is not plastic; on the contrary, compensatory activation of the RIFG has been found in patients with left prefrontal glioma (Miotto et al., 2014). Additionally, when the LIFG was damaged, other regions of the surviving language network (including the left posterior temporal lobe and perilesional region) are also known to be compensatory-activated (Stockert et al., 2020). Compensation of the RIFG may not be easily observed when various regions are widely involved in compensation. Another explanation is that the brain reorganization caused by the tumor is progressive (Desmurget et al., 2007), so RIFG reorganization may not be easily observed in patients with left frontal glioma in the early compensatory stage. In conclusion, our study demonstrates the importance of the LIFG in frontal compensation and provides evidence for the intraoperative protection of the frontal function. In the future, the language function of patients with glioma should be evaluated to further prove the role of the IFG compensation in the protection of the language function.

The OFC is a key region associated with executive functions and emotions, whereas the medial and lateral regions encode reward/pleasure and punishment/unhappiness, respectively (Rudebeck and Rich, 2018; Rolls, 2019). Previous studies have reported the plasticity of the OFC (Vik et al., 2019; Babu et al., 2020). In our study, patients did not show significant changes in executive functions, which we hypothesized were related to the structural reorganization of the OFC. Additionally, further scales analysis is necessary to explore the behavioral significance of the structural and functional reorganization. The OFC is not isolated; on the contrary, it is directly connected to the IFG, especially on the right side (Heather Hsu et al., 2020). Compared with the IFG, the function of the OFC is considered to be symmetrically organized between the two hemispheres (Stalnaker et al., 2015), which we hypothesize allows observable compensations to occur on both sides. In our study, the vast majority of patients (99 out of 101) show no mental or psychological changes, which demonstrates the physiological significance of the structural compensatory in OFC. In future studies, the cognitive functions and mental statuses of patients should be quantitatively assessed to confirm the role of OFC compensation in the protection of the brain function.

### Study limitations and future directions

The plasticity of the brain is mechanistically very complex. Our study has demonstrated the overall contralateral plasticity of the brain in patients with frontal glioma. The next research should focus on the network-level structural and functional reorganization. Additionally, the advantages of 3DT1, fMRI (including task-state fMRI and rsfMRI) and DTI should be combined to comprehensively evaluate the structural and functional reorganization of the brain in glioma patients. It is known that the callosum changes after brain injury to promote the contralateral reorganization. However, the changes in other WM fibers and their contribution to GM reorganization are still unknown. Our study explains why patients did not develop the expected functional deficit before surgery, but some patients developed progressive symptoms, such as hemiplegia and hemidysesthesia, after discharge. Therefore, longitudinal radiographic follow-up is necessary to reveal postoperative brain reorganization. It should be noted that isolated frontal gliomas are rare. Due to the close proximity between the frontal lobe and the insula, frontal tumors often invade the insula, so we included the insula as the ROI. In addition, the number of patients per WHO grade was very limited, resulting in a high degree of patient heterogeneity. In future studies, we will recruit more patients with frontal lobe only gliomas to improve these two problems.

## Conclusion

Our study provides evidence for the contralateral structural and functional reorganization in patients with frontal glioma. We have demonstrated that the IFG and OFC are two key points of the frontal compensation, and the structural compensation in the LIFG of patients with right frontal glioma synergizes with the functional compensation. Our results help to understand why patients with unilateral frontal glioma can embody functional balance even if the tumor has invaded an important functional region. Accordingly, our findings enrich the theory of brain plasticity in patients with glioma and provide new evidence for brain function protection while maximizing tumor removal.

## Data availability statement

The raw data supporting the conclusions of this article will be made available by the authors, without undue reservation.

## Author contributions

JC, HG, and YL: conception and design. HG, ZY, XH, WQ, KY, DL, and YZ: acquisition of data. HG, JC, YL, YZ, and WQ: analysis and interpretation of data. HG, JC, and DL: drafting the article. DL, JC, SC, and YZ: critically revising the article. YL, XH, SC, KY, and YZ: review submitted version of manuscript. JC: approved the final version of the manuscript on behalf of all authors. HL, HG, and ZY: statistical analysis. HL: administrative, technical, and material support. HL and JC: study supervision. All authors contributed to the article and approved the submitted version.

## Footnotes

1
http://www.rfmri.org

2
http://www.fil.ion.ucl.ac.uk/spm

3
http://www.mathworks.com/products/matlab/

4
https://www.nitrc.org/forum/forum.php?forum_id=1659

## Abbreviations

GMV: gray-matter volume
ALFF: amplitude of low-frequency fluctuation
VBM: voxel-based morphometry
MRI: magnetic resonance imaging
fMRI: functional MRI
GM: gray matter
HC: healthy control
3D: three-dimensional
TE: echo time
TR: repetition time
TI: time inversion
FA: flip angle
rsfMRI: resting-state fMRI
FOV: fields of view
WM: white matter
CSF: cerebrospinal fluid
TIV: total intracranial volume
ROI: region of interest
TFCE-FWE: threshold-free cluster enhancement family-wise error
SOG: superior occipital gyrus
ROrG: right orbital gyrus
RGR: right gyrus rectus
LOrG: left orbital gyrus
LGR: left gyrus rectus
LMFG: left middle frontal gyrus
LIFG: left inferior frontal gyrus
OFC: orbitofrontal cortex
RIFG: right inferior frontal glioma.
